# Comparative Analysis of Real-World Clinical Outcomes of a Novel Pulsed Field Ablation System for Pulmonary Vein Isolation: The Prospective CIRCLE-PVI Study

**DOI:** 10.3390/jcm13237040

**Published:** 2024-11-21

**Authors:** Lyuboslav Katov, Yannick Teumer, Carlo Bothner, Wolfgang Rottbauer, Karolina Weinmann-Emhardt

**Affiliations:** Department of Cardiology, Ulm University Heart Center, Albert-Einstein-Allee 23, 89081 Ulm, Germany; lyuboslav.katov@uniklinik-ulm.de (L.K.); yannick.teumer@uniklinik-ulm.de (Y.T.); carlo.bothner@uniklinik-ulm.de (C.B.); wolfgang.rottbauer@uniklinik-ulm.de (W.R.)

**Keywords:** atrial fibrillation, pulsed field ablation, pulmonary vein isolation, circular pulsed field ablation catheter

## Abstract

**Background:** Pulsed field ablation (PFA) represents a novel non-thermal approach for treating atrial fibrillation (AF) through pulmonary vein isolation (PVI). By utilizing irreversible electroporation, PFA creates lesions with minimal impact on adjacent tissues. This study investigates the procedural outcomes and safety of a novel circular PFA catheter in comparison to an established PFA system in a real-world clinical setting. **Methods**: This prospective, single-center study enrolled 125 consecutive patients with symptomatic paroxysmal or persistent AF undergoing first-time PVI with PFA at Ulm University Heart Center. Twenty-five patients underwent PFA PVI using a novel PFA system (PulseSelect^TM^, Medtronic, Dublin, Ireland) which incorporates a new circular catheter design and additional features such as ECG-triggered energy application and phrenic nerve capture testing. In comparison, 100 patients were treated using the established PFA system (Farapulse^TM^, Boston Scientific, Marlborough, MA, USA). **Results**: Acute PVI was achieved in 100% of the patients. Procedure duration, total left atrial (LA) time and fluoroscopy time remained comparable between both groups. The total number of energy deliveries was higher with the novel circular PFA catheter (34.0 vs. 32.0; *p* < 0.001). No procedure-related complications, including pericardial tamponade, phrenic nerve injury, atrial-esophageal fistula, vascular complications, embolisms, malignant cardiac arrhythmias, or coronary spasms were observed. **Conclusions**: The novel and the established PFA systems demonstrated comparable results in terms of procedure duration, fluoroscopy time, and LA time. In the hands of experienced operators, the novel circular PFA system enables an effective, consistent, and safe approach to successful PFA PVI.

## 1. Introduction

Catheter ablation has emerged as a highly effective treatment for patients with symptomatic, drug-refractory atrial fibrillation (AF), providing an alternative to pharmacological approaches [1,2]. Despite its efficacy, conventional thermal ablation techniques carry the risk of specific procedural complications such as esophageal injury and phrenic nerve injury [3,4,5]. Pulsed field ablation (PFA) offers a novel, non-thermal technique that creates lesions through irreversible electroporation, a process that disrupts cell membranes without significant thermal damage to surrounding tissues [6,7,8,9]. This method has demonstrated potential in achieving pulmonary vein isolation (PVI) and with proving non-inferiority for PFA compared to thermal PVI modalities in the ADVENT trial [10]. Recent clinical studies, such as the PULSED AF pivotal trial, have additionally highlighted the efficacy and safety of PFA, demonstrating its ability to achieve PVI efficiently, even in first-time users [9,11].

In the context of evolving ablation technologies, it is essential to evaluate the procedural outcomes and safety of new methods in real-world settings. Our study aims to analyze these parameters for the novel PFA system based on the first 25 cases performed at our center and compared to an established PFA system. By focusing on procedural efficiency and safety, this analysis seeks to provide practical insights into the application of the novel PFA system in a clinical environment.

## 2. Materials and Methods

### 2.1. Study Cohort

In our prospective study, we enrolled 125 consecutive patients who underwent first-time PFA PVI at Ulm University Heart Center between December 2023 and September 2024. A total of 25 patients were treated with a novel circular PFA catheter (PulseSelect^TM^, Medtronic, Dublin, Ireland) and compared to 100 patients that were treated with an established PFA system (FaraPulse^TM^, Boston Scientific, Marlborough, MA, USA). Inclusion criteria were symptomatic paroxysmal or persistent AF with a planned first-time PVI. Patients with long-standing persistent AF, other left atrial (LA) arrhythmias requiring additional procedures such as 3D mapping and radiofrequency (RF) ablation, or prior LA ablations were excluded from this study. The data were collected prospectively as part of the ATRIUM registry (German Clinical Trials Register-ID: DRKS00013013). All participants provided written informed consent. This research received approval from the local Ethics Committee of Ulm University and adheres to the principles outlined in the Declaration of Helsinki.

### 2.2. Periprocedural Management, Ablation Procedure, and Postprocedural Management

Patients remained on uninterrupted oral anticoagulation for the procedure, and for those at high thromboembolic risk, oral anticoagulation was administered for a minimum of three weeks before the ablation procedure [1]. No preprocedural cardiac imaging was performed. Deep sedation was induced using our standard protocol, starting with a midazolam bolus followed by a continuous infusion of propofol to prevent any accidental movement of the patient [12]. The intervention was performed with the patient breathing spontaneously, supported by upper airway assistance using nasopharyngeal or oropharyngeal tubes. The level of consciousness was regularly assessed throughout the procedure [13]. Transesophageal echocardiography (TEE) (Philips CX50 ultrasound system, with a Philips X7 TEE probe, Philips, Amsterdam, The Netherlands) was used to exclude the presence of atrial thrombus [12,14,15]. Afterwards an esophageal multi-electrode temperature probe (S-Cath, Circa Scientific LLC, Englewood, CO, USA) was transnasally positioned at the level of the left atrium. Access to the left atrium was achieved via right vena femoralis communis, vena cava inferior, and the right atrium, followed by a transseptal puncture (TSP). This was facilitated by a dual puncture of the right femoral vein under ultrasound guidance, followed by the insertion of a steerable 10-polar coronary sinus catheter (Inquiry™ Steerable Diagnostic Catheter, Abbott, North Chicago, IL, USA) to monitor intracardiac signals.

Using a non-steerable transseptal sheath and a transseptal needle (CardiaGuide™ non-steerable transseptal sheath, Fixed Sheath, Biosense Webster, Irvine, CA, USA, and HeartSpan™ Transseptal Needle, Biosense Webster, Irvine, CA, USA) the TSP was performed under the guidance of fluoroscopy and transesophageal echocardiography. After successful access to the left atrium, an LA pressure waveform was recorded. Using TEE and fluoroscopic guidance, a coronary guidewire (Balance Heavyweight™, Abbott, North Chicago, IL, USA) was advanced into the left superior pulmonary vein (LSPV), and the non-steerable sheath was then positioned over the guidewire. Once the sheath was in a stable position, the needle, dilator, and guidewire were withdrawn [15].

Heparin and fentanyl were administered upon entry into the left atrium to maintain an activated clotting time between 300 and 350 s. An additional dose of atropine was given to prevent bradycardia due to vagal stimulation [16]. Selective pulmonary vein (PV) angiography was performed, and the PVI procedure continued according to specific protocol, as described below.

Following the completion of the ablation, the equipment was withdrawn into the right atrium and removed. The puncture site was closed with a figure-of-eight suture, and transthoracic echocardiography was conducted to rule out complications such as pericardial effusion. The patient’s neurological status was evaluated upon recovery from sedation. Oral anticoagulation was maintained without interruption, with the duration determined by the CHA_2_DS_2_-VA score and set to at least two months post-ablation [1].

### 2.3. Pulsed Field Ablation Protocol with the Novel Circular PFA Catheter

**Figure 1:**
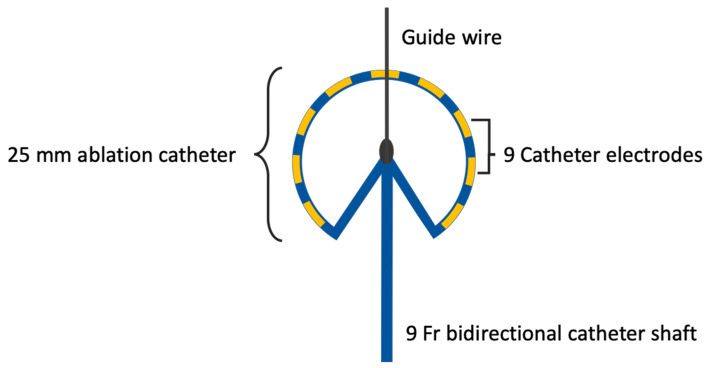
Depiction of the novel circular PFA catheter with a 25 mm spiral loop, featuring 9 electrodes (yellow), and attached to a 9 Fr bidirectional steerable catheter shaft. The array is 20° tilted to optimize tissue contact for PVI. Fr, French; PFA, Pulsed field ablation; PVI, pulmonary vein isolation.

After performing the PV angiography, the non-steerable transseptal sheath was replaced by a steerable PFA sheath (FlexCath Contour™, Medtronic, Dublin, Ireland). This was advanced with guidance from an extra-stiff guidewire (Amplatz Support Wire Guide^TM^, Cook Group, Bloomington, IN, USA). Following this, the novel circular PFA catheter was advanced into the LSPV via the guide wire (InQwire^TM^, Merit Medical, South Jordan, UT, USA). The advancement and precise positioning of the PFA catheter were monitored using fluoroscopy in the anteroposterior (AP) view as well as in the left anterior oblique (LAO) at 40° angles and in the right anterior oblique (RAO) at 30° angles. Prior ablation electrical cardioversion was performed, to convert patients to sinus rhythm, if needed. The novel circular PFA catheter was available in one size of 25 mm and features a total of 9 electrodes (Figure 1) [17].

**Figure 2:**
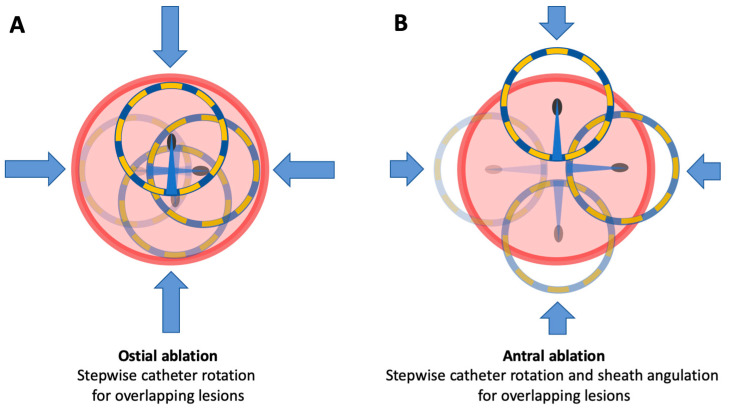
Depiction of the lesion generation with the novel circular PFA catheter at the PV. (**A**) Ostial approach using a circular PFA catheter in a horseshoe configuration. The catheter is rotated to deliver energy at superior, anterior, inferior, and posterior positions (blue arrows), forming an ostial lesion. (**B**) Antral approach with the catheter rotated as in (**A**). The sheath is angled to create an additional circumferential lesion for antral isolation. PFA, pulsed field ablation; PV, pulmonary vein.

We performed at least eight 4 s energy deliveries (EDs) per PV: four at the ostium and four at the antrum of the vein. To create an ostial lesion, the circular PFA catheter, which is configured in a horseshoe shape, is rotated four times around its axis to deliver energy at the superior, anterior, inferior, and posterior positions. Accurate positioning at the ostium is confirmed by an evenly distributed deformation of the PFA catheter upon wall contact, ensuring that ablation is confined to the targeted area without extending too deeply into the PV. Before each rotation, the catheter is slightly retracted from the PV ostium, then advanced again following rotation to maintain consistent wall contact. This technique helps prevent unintended deformation or potential catheter damage. During each rotation, the open end of the horseshoe-shaped catheter is oriented toward the center. To achieve antral isolation, in addition to the catheter rotation described above, the sheath is angled, thereby enabling the generation of an additional circumferential lesion. The guide wire, securely positioned in the PV, provides stability and prevents any displacement of the PFA catheter during angulation or rotation at each position (Figure 2).

With the aid of catheter rotations, we checked for residual signals (entrance block) after the routine EDs. Following this, stimulation was performed from the vein to confirm an exit block. If there were no entrance or exit blocks, additional EDs were performed until entrance and exit blocks were achieved.

**Figure 3:**
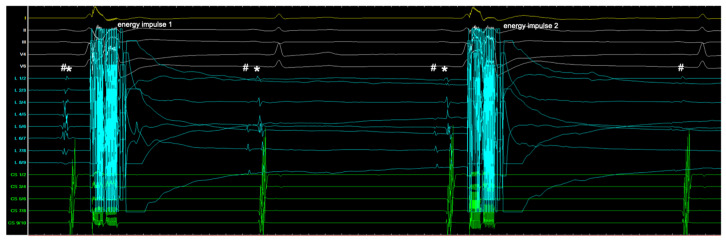
Depiction of two of four energy impulses of one ED with real-time PVI. Recordings: 12-channel surface ECG (yellow/white), PFA catheter signals (turquoise), and coronary sinus catheter signals (green). Shown are the first two energy impulses (1 and 2) of an ED from the circular PFA catheter. Before application 1: Pulmonary vein signal overlaid with atrial far-field (#). After application 1: Dissociation of far-field (#) and pulmonary vein signal (*) indicating partial isolation. After application 2: Complete vein isolation; pulmonary vein signal (*) absent, only far-field (#) remains. Paper speed 100 mm/s; ED, energy delivery; PFA, pulsed field ablation; PVI, pulmonary vein isolation.

One ED consists of four energy impulses, each followed by a one-second pause. This relatively slow energy delivery allows for real-time PVI monitoring, as the signals gradually disappear with each application, providing sufficient time for one to two signals to be observed between energy impulses (Figure 3). The evaluation of signals and the ability to achieve real-time PVI are similar to those seen with cryoballoon ablation.

All EDs were triggered by the electrocardiogram (ECG-triggered). The esophageal temperature was monitored during every ED. A threshold for interrupting the ED was set at an esophageal temperature of 40 °C. Prior to ablation at the right PVs, stimulation was performed from the ablation site to exclude phrenic nerve capture. If phrenic nerve capture was present, the catheter was relocated, if possible, to a more antral position, and a new test delivery was performed.

### 2.4. Pulsed Field Ablation Protocol with the Established PFA Catheter

**Figure 4:**
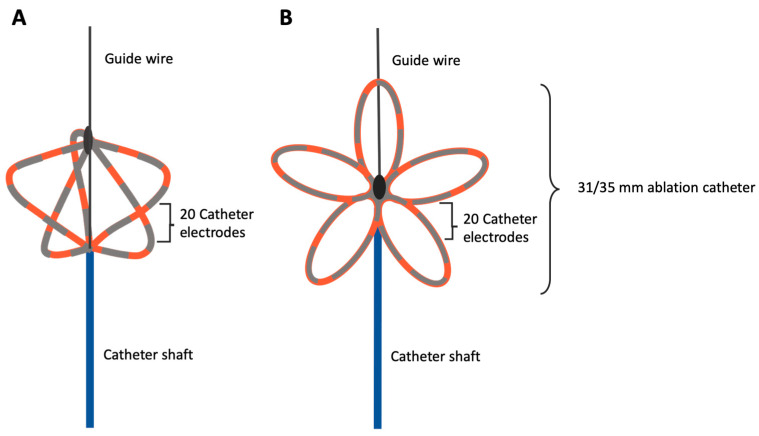
Depiction of the used PFA catheter in the basket (**A**) and the flower configuration (**B**). PFA, pulsed field ablation.

For the PFA PVI with the established PFA catheter, a steerable PFA sheath (Faradrive^TM^, Boston Scientific, Marlborough, MA, USA) was positioned in the left atrium, guided by an extra-stiff guidewire. Subsequently, the PFA catheter (Farawave^TM^, Boston Scientific, Marlborough, MA, USA) was navigated to the LSPV (Figure 4).

**Figure 5:**
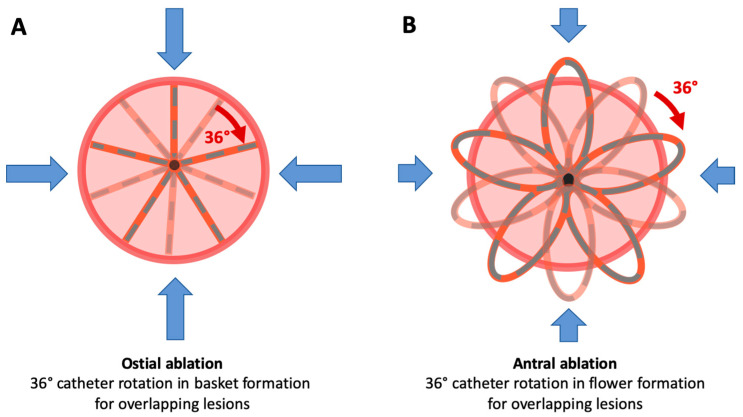
Depiction of the PVI with the established PFA catheter. (**A**) Positioning of the basket configuration at the vein, with a 36° rotation for ostial lesion creation in a superior, anterior, inferior, and posterior direction (blue arrows). (**B**) Positioning of the flower configuration at the vein, with a 36° rotation for antral lesion creation. PFA, pulsed field ablation; PVI, pulmonary vein isolation.

Detailed information regarding the PFA catheter configuration and the specific ablation protocol has been described before [11]. To achieve isolation of the PV, a minimum of eight 2.5 s EDs were administered using the established PFA system: four ostial and four antral EDs. For creating an ostial lesion, the PFA system was positioned at the vein in the basket configuration, followed by two EDs. The device was then rotated by 36°, and an additional two EDs were delivered. For antral PVI, the system was retracted into the flower configuration, which has a maximum diameter of 31 or 35 mm, depending on the catheter selection. In this configuration, the catheter was advanced to the vein over a wire. Two EDs were delivered, followed by a 36° rotation and two more EDs, ensuring overlapping isolation (Figure 5).

**Figure 6:**
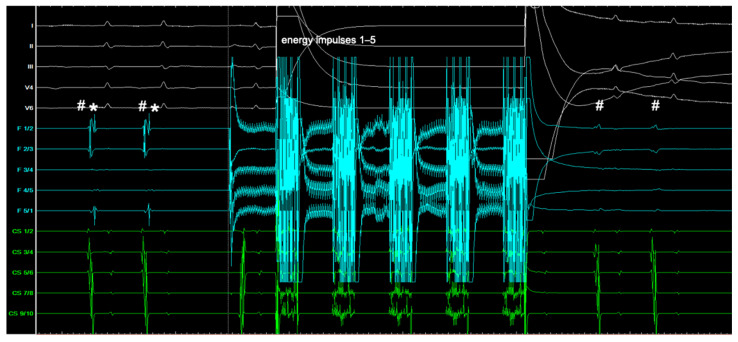
Depiction of five energy impulses of one ED with PVI. Recordings: 12-channel surface ECG (white), PFA catheter signals (turquoise), and coronary sinus catheter signals (green). Shown are all five energy impulses (1–5) of one ED of the established PFA catheter. Before application 1: Pulmonary vein signal overlaid with atrial far-field (#). After five consecutive energy impulses: Complete vein isolation; pulmonary vein signal (*) absent, only far-field (#) remains. A total of 100 mm/s paper speed: ED, energy delivery; PFA, pulsed field ablation; PVI, pulmonary vein isolation.

One ED, using the established PFA system, consists of five energy impulses delivered consecutively without any significant pause in a cumulative 2.5 s. The observation of real-time PVI in between the energy impulses is not possible (Figure 6).

### 2.5. Comparison Between Both Systems

The ablation mechanism employed by both systems is electroporation, utilizing non-thermal energy. The systems differ in catheter size and configuration, as well as the number of electrodes and the voltage applied per ED. New features in the novel circular PFA catheter include ECG-triggered ablation and real-time monitoring of phrenic nerve capture. Both systems are compatible with integration into 3D mapping systems. A detailed comparison of both systems is presented in Table 1.

### 2.6. Statistical Analysis

Statistical analysis was performed using SPSS**^®^** Statistics (version 29.0.1.0, IBM, Armonk, New York, NY, USA). Categorical variables were assessed using the Chi-Square or Fisher’s exact test. The Mann–Whitney U test was performed for continuous variables. The results were reported as median values along with interquartile ranges (IQRs). A *p*-value of < 0.05 was considered statistically significant.

## 3. Results

The median age of the participants was 68.0 (62.0; 74.5) years. Of the participants, 52.0 (41.6%) were female and the median BMI was 27.1 (24.3; 30.2) kg/m^2^. The median left ventricular ejection fraction was normal to mildly reduced (60.0 (50.5; 64.5)%). The median left atrial diameter was 4.4 (3.9; 4.9) cm (LAVI 36.0 (27.5; 46.6) mL/m^2^). A detailed overview of the baseline characteristics is provided in Table 2.

There were no significant differences between the systems in terms of procedure duration (54.0 vs. 62.0, *p* 0.14), fluoroscopy time (16.6 vs. 14.9 min, *p* 0.18), and total LA time (37.0 vs. 38.5 min, *p* 0.35) (Table 3).

Both the established and the novel circular PFA catheters showed a 100% success rate in acute PVI. The novel circular PFA catheter required significantly more EDs for LSPV, LIPV, RIPV, and RSPV isolation compared to the established PFA catheter. Additionally, the total EDs per patient was higher with the novel circular PFA catheter (*p* < 0.001) (Table 4).

In 13 patients (13.0%), the 35 mm device from the established PFA system was selected for PVI due to a common ostium or large-diameter PV observed on PV angiography. In the remaining 87 patients (87%), the standard 31 mm PFA catheter was used.

**Figure 7:**
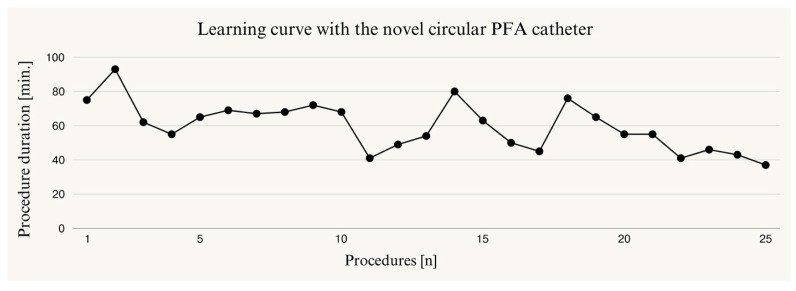
Depiction of the learning curve in the cohort treated with the novel circular PFA catheter.

During the learning curve with the novel circular PFA catheter, a significant difference in procedural duration was observed between the first and last 5 patients treated with the system (*p* = 0.009) (Figure 7).

No complications were observed in this study (Table 5). In none of the procedures performed with the novel ablation catheter was PFA catheter repositioning required due to phrenic nerve capture following the test pulse. Additionally, in 25% of the patients from the same cohort, esophageal temperature monitoring was performed using a probe, but no relevant temperature rise above 37 °C was observed.

## 4. Discussion

For years, catheter ablation has been the standard treatment for symptomatic atrial fibrillation refractory to antiarrhythmic drug therapy, demonstrating favorable outcomes, particularly in patients with heart failure and reduced left ventricular ejection fraction [1]. PFA has ushered in a new era in ablation therapy, primarily due to its tissue-selective properties. In a randomized trial by Osmancik et al., PFA was found to cause more extensive myocardial damage than point-by-point RF ablation. Despite this, PFA was associated with a lower inflammatory response, a finding also supported by previous animal studies [18,19,20]. In the ADVENT trial, Reddy et al. demonstrated that PFA is non-inferior to thermal modalities for PVI [10]. Moreover, the PFA PVI demonstrated a rapid learning curve in first-time users and a shorter procedure duration compared to established thermal techniques [11]. Despite these developments, to our knowledge, there has been no direct comparison between the two latest PFA systems worldwide: the established PFA catheter and the novel circular PFA catheter. In our study, we compared the two PFA systems among first-time experienced users regarding efficacy and safety.

### 4.1. Procedural Performance

Comparison of procedural data between the two systems reveals no significant difference in overall procedure duration. LA times were also similar, suggesting that the procedures with both PFA systems were performed at a comparable pace. The comparable duration indicates that the novel circular PFA system is as efficient as the established one. Fluoroscopy times are similar as well, reinforcing the conclusion that both systems are equally effective without any additional time required for the novel circular catheter, neither for ablation nor for catheter preparation.

Regarding ED, both total and per vein, the novel circular mapping catheter requires significantly more energy applications compared to the established system. Both systems have a recommended minimum of four ostial and four antral energy applications. The increased number of energy applications with the novel circular system is likely due to the different catheter configuration. The established PFA system employs two configurations: basket and flower. Each configuration rotates 36° after two EDs to ensure a continuous circumferential ablation, with the system rotating around its axis to create reproducible and stable ostial and antral isolation lines. This typically results in minimal residual signals after rotation within this stable radius.

In contrast, the novel circular PFA system is horseshoe-shaped. The initial four ostial lesions are created similarly to the established system, by rotating the device around its axis to achieve a circular lesion. However, the antral workflow differs significantly. To create the antral lesion, the catheter’s circular portion is rotated outward, with the system’s radius adjusted by angulating the sheath in superior, anterior, inferior, and posterior directions, as the device diameter cannot be varied. This final step often requires additional energy applications to address residual signals in specific regions, such as superoanterior, inferoanterior, superoposterior, and inferoposterior, to ensure a complete circular lesion. In summary, the differences in voltage per ED, delivery mode, and catheter configuration play crucial roles. The established system’s adjustable diameters and simple rotation around its axis contrast with the novel circular system, which relies on sheath angulation to expand the ablation diameter, leading to more energy applications to achieve a complete lesion. The long-term appearance of the lesions will need to be evaluated in follow-up studies and during re-PVI procedures.

The established catheter system offers the advantage of tailoring the catheter size to the patient’s specific anatomy, particularly based on PV angiography, accommodating the size of the PVs and left atrium. In its basket configuration, the device can be adjusted to match the PV diameter; it can be elongated for smaller veins to facilitate entry and shortened for larger veins to maintain wall contact, allowing for accurate PV signal detection and exit block testing. For antral lesions, the device is available in two sizes, with a maximum diameter of 31 mm or 35 mm in the flower configuration. This allows for the selection of a larger device when pulmonary vein (PV) angiography indicates a larger diameter, which was required in 13.0% of cases. In contrast, the novel circular PFA system is only available in a single, non-variable size. While the design allows some flexibility through sheath rotation, enabling greater angulation to cover larger PV ostia, this often requires additional EDs per ostium to achieve complete coverage. To determine PV signals and test for exit block, the device must be elongated to enter the vein, resulting in a corkscrew configuration. However, this configuration may not cover the entire PV circumference, potentially leading to gaps in the ablation line. Additionally, the catheter, when stretched, extends further into the PV, placing part of it within non-conductive tissue, which may compromise the accuracy of exit block testing.

The novel circular PFA system is better suited for visualizing the entrance block with real-time PVI, providing more precise and immediate monitoring of PVI. The established system uses 2000 V ED with very short application times (five energy impulses in 2.5 s), resulting in the elimination of PV signals in nearly all cases after the initial series of energy applications. In contrast, the novel system delivers lower voltage per application (1500 V) with more spaced-out energy impulses (four energy impulses in 4 s), allowing for real-time PVI as the signals gradually disappear, similar to cryoballoon ablation. The separation of atrial far-field signals from pulmonary vein signals can be observed, confirming real-time PVI. In summary, the observed consecutive migration and eventual disappearance of the atrial signals are attributed to the longer distance between the energy impulses, along with lower voltage delivery, which also results in a significantly higher number of EDs in patients treated with the novel circular PFA system. Thus, similar to cryoablation pulmonary vein isolation, the focus of the operator remains on the signals within the vein. In contrast, with the established system, real-time PVI cannot be observed. After the energy delivery from five consecutive energy impulses, the vein is either isolated or not. Here, the focus is less on the electrocardiogram.

The novel circular system features a diameter comparable to that of the standard cryoballoon. However, there are notable differences between the two devices. For instance, the novel circular system offers enhanced flexibility due to its horseshoe shape and a wider range of sheath angulation. A significant drawback is, however, the lack of direct verification of wall contact, which is achievable during cryoablation through the use of contrast agent delivery. In contrast, the novel circular PFA catheter relies exclusively on fluoroscopy-guided positioning and catheter deformation following wall contact.

Another important point concerns the configuration and maneuvering of the catheter from one vein to the next. In the established system, the workflow involves transitioning the device between veins in the flower configuration with the wire fully retracted. The flower configuration offers a flat, atraumatic surface that facilitates maneuvering the device through the atrium, thereby minimizing the risk of perforation. In contrast, the novel circular device features a small tip where the wire exits, and to avoid perforation, the wire should never be fully retracted into the device. In smaller atria or in challenging locations such as the RIPV, where space is limited, the wire can restrict the catheter’s maneuverability and make vein cannulation more difficult. Additionally, accidental retraction of the wire could increase the risk of perforation.

The learning curve of the novel PFA system demonstrates a significant reduction in procedure duration within the first 25 cases, with a plateau observed towards the end of this study. This indicates that the novel circular PFA system has a short learning curve and is readily applicable by experienced electrophysiologists, similar to the learning curve observed with the established PFA system during first-time use [11]. As previously reported by Velagic, the learning curve for cryoballoon ablation is notably shorter compared to point-by-point RF ablation. In our opinion, the comparable procedural times observed in our study may be attributed to the similarities in workflow and handling between PFA systems and cryoablation techniques. This suggests that experienced cryoablation users may adapt more readily to PFA systems, thereby enhancing procedural efficiency and facilitating a smoother transition between modalities. The familiarity with workflow dynamics and technical maneuvers likely contributes to the observed procedural outcomes and rapid learning curve [21].

### 4.2. Procedural Safety

Regarding safety, no complications were observed in either group. Specifically, there were no instances of pericardial effusion/tamponade, phrenic nerve injury, atrial-esophageal fistula, vascular complications, stroke or transient ischemic attacks, malignant cardiac arrhythmias, or coronary spasms. Additionally, in the study cohort, esophageal temperature was partially monitored, with no significant increase noted. Furthermore, the novel circular device includes the capability for phrenic nerve testing. Prior to ablation of the right PVs the phrenic nerve can be stimulated from the ablations site, and if there is a capture the catheter can be replaced, which helps prevent phrenic nerve injury during the procedure. As previously reported in the one-year outcomes from the MANIFEST-PF Registry, persistent phrenic nerve injury was observed in 0.06% of cases, and transient injury occurred in 0.4%, during ablation with the established PFA catheter [22]. In our study, no instances of phrenic nerve injury were observed in both groups.

Another new feature of the novel PFA system is ECG triggering, which prevents ED during the ventricular refractory phase, adding an extra layer of security against the induction of malignant arrhythmias [17]. However, interruptions in ablation delivery were occasionally noted due to reference signal loss, an issue resolved by changing the reference electrode. Neither the established nor the novel PFA system induced malignant arrhythmias such as ventricular tachycardia or ventricular fibrillation. Both systems appear to be safe in their ED.

### 4.3. Limitations

This study is limited by its relatively small sample size, especially in the group treated with the novel circular PFA catheter. The limited number of patients is due to the device being available for only a short period. Larger, multicenter studies are required to validate these findings and explore the long-term clinical implications of using different PFA systems.

## 5. Conclusions

Regardless of the ablation system used, PVI was successfully achieved in all patients. Both systems demonstrated the same procedure duration, fluoroscopy time, and LA time. The learning curve for both systems was comparable and fast. The established PFA system provided highly stable handling and consistent energy application due to its fixed device radius. In contrast, the novel circular PFA system offered greater flexibility through its horseshoe design and sheath angulation, albeit with a less stable radius for EDs. No procedural complications were observed in either group. In the hands of experienced operators, the novel circular PFA system enables an effective, consistent and safe approach to successful PFA PVI.

## Figures and Tables

**Table 1:** Comparison of the differences between both systems.

System Characteristics	Established PFA Catheter	Novel Circular PFA Catheter
Mechanism of ablation	Selective cell electroporation	Selective cell electroporation
Form of energy	Non-thermal	Non-thermal
Device diameter	31 mm or 35 mm	25 mm
Device configuration during ED	Routinely ‘basket’ and ‘flower’, as well as every position in-between	Circular
ED	At least 8 EDs per vein	At least 8 EDs per vein
Voltage pro ED	2000	1500
Signals monitoring in the PVs	Via 20 electrodes	Via 9 electrodes
ECG-trigger	No	Yes
Phrenicus nerve capture test	No	Yes
Possibility to integrate in 3D system	Yes	Yes

ECG, electrocardiogram; ED, energy delivery; PFA, pulsed field ablation; PV, pulmonary vein.

**Table 2:** Baseline characteristics of the patients treated with both systems.

Baseline Characteristics	All Patients (*n* = 125)	Established PFA Catheter (*n* = 100)	Novel Circular PFA Catheter (*n* = 25)	*p*-Value
Age [years], median (IQR)	68.0 (62.0; 74.5)	68.0 (62.0; 74.7)	69.0 (63.5; 75.0)	0.66
Female, *n* (%)	52.0 (41.6)	39.0 (39.0)	13.0 (52.0)	0.24
BMI [kg/m^2^], median (IQR)	27.1 (24.3; 30.2)	27.1 (24.3; 30.3)	26.4 (24.2; 30.2)	0.51
CHA_2_DS_2_-VA score, median (IQR)	3.0 (2.0; 4.0)	3.0 (2.0; 4.0)	2.0 (1.0; 4.0)	0.05
LA diameter [cm], median (IQR)	4.4 (3.9; 4.9)	4.4 (3.9; 4.9)	4.5 (3.8; 4.7)	0.82
LAVI [mL/m^2^], median (IQR)	36.0 (27.5; 46.6)	37.8 (27.8; 48.0)	32.6 (20.9; 39.3)	0.10
LVEF (%), median (IQR)	60.0 (50.5; 64.5)	58.0 (49.2; 64.0)	62.0 (56.0; 66.5)	0.06
Hypertension, *n* (%)	99.0 (79.2)	83.0 (83.0)	16.0 (64.0)	0.04
Diabetes mellitus, *n* (%)	20.0 (16.0)	17.0 (17.0)	3.0 (12.0)	0.76
Hyperlipoproteinemia, *n* (%)	90.0 (72.0)	74.0 (74.0)	16.0 (64.0)	0.32
Coronary artery disease, *n* (%)	52.0 (41.6)	42.0 (42.0)	10.0 (40.0)	0.86
OSA, *n* (%)	6.0 (4.8)	4.0 (4.0)	2.0 (8.0)	0.34
Prior Stroke/TIA, *n* (%)	10.0 (8.0)	8.0 (8.0)	2.0 (8.0)	1.00

BMI, body mass index; IQR, interquartile range; LA, left atrium; LAVI, left atrial volume index; LVEF, left ventricular ejection fraction; OSA, obstructive sleep apnea; PFA, pulsed field ablation; TIA, transient ischemic attack.

**Table 3:** Procedure characteristics of the patients treated with both systems.

Procedure Characteristics	All Patients (*n* = 125)	Established PFA Catheter (*n* = 100)	Novel Circular PFA Catheter (*n* = 25)	*p*-Value
Procedure duration [minutes], median (IQR)	55.0 (43.5; 66.0)	54.0 (42.2; 64.7)	62.0 (47.5; 68.5)	0.14
Total LA time [minutes], median (IQR)	37.0 (30.0; 48.2)	37.0 (29.0; 49.0)	38.5 (34.2; 44.5)	0.35
Fluoroscopy time [minutes], median (IQR)	16.1 (12.7; 19.4)	16.6 (12.6; 21.0)	14.9 (13.0; 17.5)	0.18
LA Pressure [mmHg], median (IQR)	10.0 (6.0; 13.0)	9.0 (6.0; 13.0)	10.0 (7.7; 15.0)	0.14

IQR, interquartile range; LA, left atrium; PFA, pulsed field ablation.

**Table 4:** Ablation parameters of the patients treated with both systems.

Ablation Parameters	Established PFA Catheter (*n* = 100)	Novel Circular PFA Catheter (*n* = 25)	*p*-Value
Acute PVI, *n* (%)	100 (100)	25 (100)	1.0
ED LSPV, median (IQR)	8.0 (8.0; 8.0)	9.0 (8.0; 12.0)	<0.001
ED LIPV, median (IQR)	8.0 (8.0; 8.0)	8.0 (8.0; 8.0)	<0.001
ED RIPV, median (IQR)	8.0 (8.0; 8.0)	8.0 (8.0; 8.0)	0.03
ED RSPV, median (IQR)	8.0 (8.0; 8.0)	8.0 (8.0; 9.0)	0.01
ED LCPV, median (IQR)	16.0 (8.0; 16.0)	14.5 (13.0; 16.0)	1.0
ED total per patient, median (IQR)	32.0 (32.0; 32.0)	34.0 (33.0; 36.5)	<0.001

EDs, energy deliveries; IQR, interquartile range; LCPV, left common pulmonary vein; LIPV, left inferior pulmonary vein; LSPV, left superior pulmonary vein; PVI; pulmonary vein isolation; RIPV, right inferior pulmonary vein; RSPV, right superior pulmonary vein.

**Table 5:** Complications in the patients treated with both systems.

Complications	Established PFA Catheter (*n* = 100)	Novel Circular PFA Catheter (*n* = 25)
Pericard effusion/tamponade, *n* (%)	0	0
Phrenicus nerve injury, *n* (%)	0	0
Atrial-esophageal fistula, *n* (%)	0	0
Vascular complications, *n* (%)	0	0
Stroke/transient ischemic attacks, *n* (%)	0	0
Malignant cardiac arrhythmias, *n* (%)	0	0
Coronary spasm noted, *n* (%)	0	0

PFA, pulsed field ablation.

## Data Availability

The data presented in this study are available on request from the authors. The data are not publicly available due to data privacy laws.
